# *Journal of Hematology & Oncology* reviewer acknowledgement 2014

**DOI:** 10.1186/s13045-015-0107-7

**Published:** 2015-03-04

**Authors:** Delong Liu, Thalyana Smith-Vikos

**Affiliations:** Department of Medicine, New York Medical College and Westchester Medical Center, Valhalla, 10595 NY USA; BioMed Central, Floor 6, 236 Gray’s Inn Road, London, WC1X 8HB UK

## Abstract

The editors of *Journal of Hematology & Oncology* would like to thank all our reviewers who have contributed to the journal in Volume 7 (2014).

**Akintunde Akinleye**

United States of America

**Ramon Alemany**

Spain

**Veerajalandhar Allareddy**

United States of America

**Lyndal Anderson**

Australia

**Peter Anderson**

United States of America

**Shunya Arari**

Japan

**Diego Arenas**

Mexico

**Tomohiro Asai**

Japan

**Michele Baccarani**

Italy

**Jonathan Back**

Switzerland

**Elisabetta Baldi**

Italy

**Frederic Baron**

Belgium

**David Bartlett**

United States of America

**Amer Beitinjaneh**

United States of America

**Leonidas Benetatos**

Greece

**Jackie Boultwood**

United Kingdom

**M Mitzi Brentani**

Brazil

**Hal Broxmeyer**

United States of America

**Davide Calebiro**

Germany

**Dan Canaani**

Israel

**Daniel Catovsky**

United Kingdom

**Angela Chan**

Canada

**Hong Chang**

Canada

**Andy Chase**

United Kingdom

**Guoan Chen**

United States of America

**Michelle Y.Q. Chen**

China

**Yi-Bin Chen**

United States of America

**Sreenivasulu Chintala**

United States of America

**William CS Cho**

Hong Kong

**David Chui**

United States of America

**Monika Csóka**

Hungary

**Zoran Culig**

Austria

**Nico Dantuma**

Sweden

**Naval Daver**

United States of America

**Guido David**

Belgium

**Marc De Braekeleer**

France

**Silvia Deaglio**

Italy

**Jiusheng Deng**

United States of America

**Husheng Ding**

United States of America

**Kenneth Dorshkind**

United States of America

**Sinisa Dovat**

United States of America

**Madeleine Duvic**

United States of America

**Brian Dymock**

Singapore

**Agnieszka Dzikiewicz-Krawczyk**

Poland

**Andreas Eisenreich**

Germany

**Monica Else**

United Kingdom

**Monika Engelhardt**

Germany

**Ferry ALM Eskens**

Netherlands

**Lucia Farina**

Italy

**Jay Fox**

United States of America

**Ota Fuchs**

Czech Republic

**Hiroshi Fukamachi**

Japan

**Muhammad Furqan**

United States of America

**Shou-Jiang Gao**

United States of America

**Enrico Garattini**

Italy

**Valter Gattei**

Italy

**Christian Gisselbrecht**

France

**Damjan Glavac**

Slovenia

**Alvaro Gonzalez**

Spain

**Finn Grey**

United Kingdom

**Manuel Grez**

Germany

**John Gribben**

United Kingdom

**Aihua Gu**

China

**Chunhua Guo**

United States of America

**Hans Haecker**

United States of America

**James Hagman**

United States of America

**Hideo Harigae**

Japan

**Claire Harrison**

United Kingdom

**Dongmei He**

China

**Jeff Holst**

Australia

**Michael Hsiao**

Taiwan

**Chung-Tsen Hsueh**

United States of America

**He Huang**

China

**Xiaojun Huang**

China

**Suming Huang**

United States of America

**Kam HUI**

Singapore

**Jung-Jyh Hung**

Taiwan

**Motoshi Ichikawa**

Japan

**Shinsuke Iida**

Japan

**Michael Ittmann**

United States of America

**Tomoo Iwakuma**

United States of America

**Yongping Jiang**

United States of America

**Zhong Jiang**

United States of America

**Zhenyu Ju**

China

**Hsueh-Fen Juan**

Taiwan

**Tamar Juven-Gershon**

Israel

**Dieter Kabelitz**

Germany

**Bin Kang**

United States of America

**Lyndal Kearney**

United Kingdom

**Sophia Khaldoyanidi**

United States of America

**Evgeny Klyuchnikov**

Germany

**Sergej Konoplev**

United States of America

**Gopal Kundu**

India

**Ann LaCasce**

United States of America

**Stephen Langabeer**

Ireland

**Alessandro Laviano**

Italy

**Hillard Lazarus**

United States of America

**John Lednicky**

United States of America

**Claudia Lengerke**

Switzerland

**Sirpa Leppä**

Finland

**Vera Levina**

United States of America

**Chengang LI**

United States of America

**Qiang Li**

United States of America

**Shaoguang Li**

United States of America

**Yangqiu Li**

China

**Patrick Lin**

United States of America

**Ke Liu**

United States of America

**Wenyu Liu**

United States of America

**Jun Liu**

United States of America

**Pengyuan Liu**

United States of America

**Luca Lo Nigro**

Italy

**Olli Lohi**

Finland

**Xiaolin Luo**

United States of America

**Nigel Mackman**

United States of America

**Daichi Maeda**

Japan

**Saradhi Mallampati**

United States of America

**Sridhar Mani**

United States of America

**Francesco Mannelli**

Italy

**John Mascarenhas**

United States of America

**Kunio Matsumoto**

Japan

**Hiromichi Matsushita**

Japan

**Anna Rita Migliaccio**

United States of America

**Ken Mills**

United Kingdom

**Chang-Ki Min**

South Korea

**Zdravko Mitrovic**

Croatia

**Yohei Miyagi**

Japan

**Helmout Modjtahedi**

United Kingdom

**L. Rhoda Molife**

United Kingdom

**Andrei Moroz**

Brazil

**Alison Moskowitz**

United States of America

**Walid Mourad**

Canada

**Marek Mraz**

Czech Republic

**Hasan Mukhtar**

United States of America

**Marta Muzio**

Italy

**Hideaki Nakajima**

Japan

**Ryotaro Nakamura**

United States of America

**David Naor**

Israel

**Javier Naval**

Spain

**Jiri Neuzil**

Australia

**Siok Bian Ng**

Singapore

**Francesco Novelli**

Italy

**Seiichi Okabe**

Japan

**Lucy Osborne**

Canada

**Thomas Pabst**

Switzerland

**Chong-xian Pan**

United States of America

**Samir Parekh**

United States of America

**Jino Park**

United States of America

**William Parker**

United States of America

**Louis Pelus**

United States of America

**Archibald Perkins**

United States of America

**Iver Petersen**

Germany

**Jan Peveling-Oberhag**

Germany

**Linda Pilarski**

Canada

**Charalampos Pontikoglou**

Greece

**Louise Purton**

Australia

**Raju Pusapati**

United States of America

**Karen Rabin**

United States of America

**John Radford**

United Kingdom

**Joseph Ragaz**

Canada

**VK Rajasekhar**

United States of America

**Pavan Mahendra Ravella**

United States of America

**Ruibao Ren**

United States of America

**Hugh Reyburn**

Spain

**Adam Rich**

United States of America

**Ingo Ringshausen**

United Kingdom

**Marthe Rizk-Rabin**

France

**Maria Rizzo**

Italy

**Pierre Rohrlich**

France

**Luisa Romao**

Portugal

**Emanuela Rosati**

Italy

**Stefan Rose-John**

Germany

**Patricia Rousselle**

France

**Jeffrey Rubnitz**

United States of America

**Ahmad Safa**

United States of America

**Kuniaki Saito**

Japan

**Ralph Salloum**

United States of America

**Brenda Sandmaier**

United States of America

**Bipin Savani**

United States of America

**Peter Sayeski**

United States of America

**Tobias Schatton**

United States of America

**Udo Schumacher**

Germany

**Heidi Schwarzenbach**

Germany

**Karen Seiter**

United States of America

**Carmine Selleri**

Italy

**Xiaojin Sha**

United States of America

**Bin Shan**

United States of America

**Mala Shanmugam**

United States of America

**Yukari Shirasugi**

Japan

**Yongqian Shu**

China

**Mitchell Smith**

United States of America

**Wenru Song**

United States of America

**Michael Spinella**

United States of America

**Eva Szegezdi**

Ireland

**Shinichiro Takahashi**

Japan

**Fumi Takahashi-Yanaga**

Japan

**Kazuhiro Tamura**

Japan

**Corrado Tarella**

Italy

**Pierfrancesco Tassone**

Italy

**Ugo Testa**

Italy

**Kensei Tobinai**

Japan

**Sanne Tonino**

Netherlands

**Jorg Tost**

France

**Ivo Touw**

Netherlands

**Mounir Trimeche**

Tunisia

**Volodymyr Tryndyak**

United States of America

**William Tse**

United States of America

**David Tulasne**

France

**Jeffrey Tyner**

United States of America

**Susumu Uchiyama**

Japan

**Anke van den Berg**

Netherlands

**Rosa Visone**

Italy

**Lydia Visser**

Netherlands

**Donghai Wang**

United States of America

**Kankan Wang**

China

**Qinhong Wang**

United States of America

**Yu Wang**

United States of America

**Erik Wiemer**

Netherlands

**Justin Wong**

Australia

**Bas Wouters**

Netherlands

**Kongming Wu**

China

**Jennifer Wu**

United States of America

**Yan Wu**

United States of America

**Dazhong Xu**

United States of America

**Hui Xu**

United States of America

**Mingjiang Xu**

United States of America

**Xiang-Xi Xu**

United States of America

**Zhen XU**

United States of America

**Xiao-Feng Yang**

United States of America

**Jilong Yang**

China

**Sherry Yang**

United States of America

**B. Hilda Ye**

United States of America

**Go Yoshida**

Japan

**M. James You**

United States of America

**Nadia Zaffaroni**

Italy

**Apostolos Zaravinos**

Sweden

**Ali Zarrin**

United States of America

**Haiyan Zhai**

United States of America

**Qing Zhang**

China

**Suping Zhang**

United States of America

**Hua Zhang**

China

**Mingjun Zhang**

United States of America

**Dekuang Zhao**

United States of America

**Meng Zhao**

United States of America

**Xiaohong Zhao**

United States of America

**Xiaohong Zheng**

United States of America

**Minghao Zhong**

United States of America

**Liang Zhou**

United States of America

**Yong Zhou**

China

**Pier Luigi Zinzani**

Italy

**Vassilis Zoumpourlis**

Greece

